# A tissue culture infectious dose-derived protocol for testing of SARS-CoV-2 neutralization of serum antibodies on adherent cells

**DOI:** 10.1016/j.xpro.2021.100824

**Published:** 2021-08-27

**Authors:** Fabio Hasler, Agathe Duda, Thomas M. Kündig, Pål Johansen

**Affiliations:** 1 Department of Dermatology, University of Zurich, 8091 Zurich, Switzerland; 2 Department of Dermatology, University Hospital Zurich, 8091 Zurich, Switzerland

**Keywords:** Cell culture, Cell-based Assays, Immunology, Microbiology

## Abstract

For a cytopathic virus such as severe acute respiratory syndrome coronavirus type 2 (SARS-CoV-2), the neutralization capacity of serum from convalescent or vaccinated persons or of therapeutic antibodies can be tested on adherent cell cultures. Here, a simple and tissue culture infectious dose-derived protocol for assessment of neutralization of SARS-CoV-2 is described. Compared with the often applied plaque-forming unit assay, the working load is lower, and fewer manipulations of the infected cultures are required. Hence, the method is safer for the personnel.

## Highlights

• The protocol describes assessment of neutralization of SARS-CoV-2 by antibodies
• Neutralization is detected by means of reduced cytopathic effect on adherent cells
• Increased safety for researchers through less manipulation of infectious cultures
• The described protocol allows for more efficient upscaling of sample testing

## Graphical abstract

**Figure:**
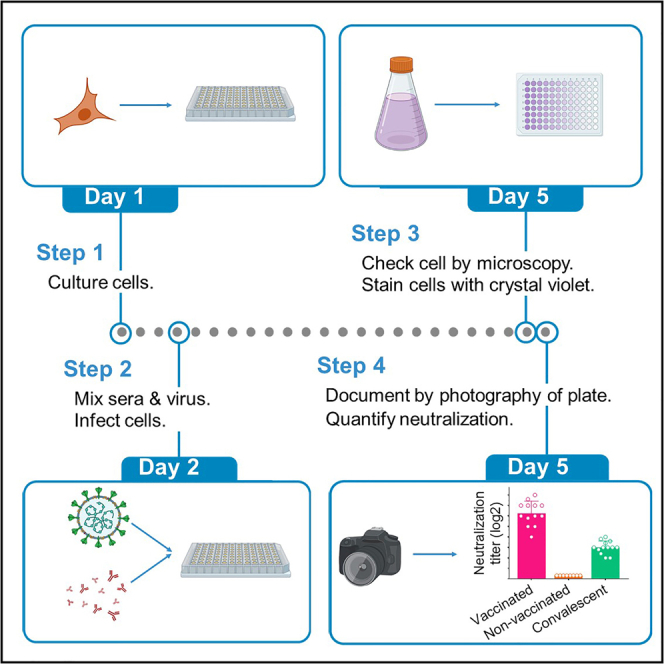

## Before you begin

This protocol applies specifically to the infection of Vero cells with SARS-CoV-2. Vero cells are kidney epithelial cells originally isolated from an African green monkey (*Cercopithecus aethiops*) and are cultured on flask as adherent cells (Ammerman et al., 2008). The basic principles of this protocol are applicable to the infection of other cell lines. Examples include the human liver carcinoma cell line HuH-7 and the human colon adenocarcinoma cell line CaCo-2, which are both susceptible to SARS-CoV-2 (Chu et al., 2020), as well as the A549 human carcinoma epithelial cells and the FT293 human embryonal kidney (HEK) cells expressing angiotensin-converting enzyme 2 (ACE2). For each cell line, the cell numbers, the medium composition, the viral numbers, and infecting incubation times may slightly vary, for which reason these parameters need to be optimized beforehand for each cell line. The indicated times are averages from working with approximately four 96-well plates. Upscaling to eight plates is feasible for handling by one researcher and is associated with an approximately 50% increase in time needed for the experimental procedures.

Prepare all the necessary ingredients (washing solution, fixation solution, staining solution) and media (DMEM #1–3) in advance. Keep the cells in culture in a separate biosafety level (BSL) 1/2 laboratory, because a BSL3 laboratory is mandatory for all steps with handling of live virus. Study carefully the internal guideline for working in a BSL3 laboratory. Make sure to have sufficient personal protective equipment available. Before doing first experiment with SARS-CoV-2, make a few experiments were you go through the protocol without using SARS-CoV-2, but with placebo (e.g., saline) or a less pathogenic but cytopathic virus (e.g., vesicular stomatitis virus, which is a BSL2 organism).

### Culturing of Vero cells

**Timing: 1 h, twice a week**

All the following procedures including the open handling of cells, culture media, and washing solutions, are performed aseptically within a biosafety hood with laminar air flow as to assure sterile cultures.

1. Add 5% Fetal Bovine Serum (FBS), 1% Minimum Essential Media (MEM) non-essential amino acids, 1% HEPES (1M; N-2-hydroxyethylpiperazine-N-2-ethane sulfonic acid), and 0.2% Normocin™ to Dulbecco's Modified Eagle Medium (DMEM) to produce the DMEM #1 medium. Store at 4°C.
2. Prepare fresh Vero cells for cell culture.
  a. Thaw one cryo tube with approximately 5×10^6^ Vero cells in 1 mL by adding 1 mL DMEM #1 medium to the tube with frozen cells. Transfer the thawed cell by using a micropipette into a 15 mL falcon tube containing another 8 mL DMEM #1 medium.
  b. Centrifuge the cells at 500 × *g* and at 18°C–22°C for 5 min.
  c. Resuspend the cells in 5 mL DMEM #1 medium and repeat the centrifugation step.
  d. Resuspend the cells in 15 mL DMEM #1. Control the cell viability by microscopy after staining a cell aliquot with trypan blue.
3. Transfer the cells to a T-150 culture flask, and incubate the flask at 37°C in a humidified and 5% CO_2_ atmosphere.
4. Split Vero cells 1:10 if 80%–100% confluence is reached, typically after ca. 6–7 days of culture for the first passage after thawing a fresh tube of cells; later, the cells grow faster with an approximately 3-days passage time.
  a. Aspirate the old culture medium from the culture flask using a serological pipette and discard the medium. Wash the cells by gently adding 5 mL Phosphate Buffered Saline (PBS) at 18°C–22°C to the flask. Aspirate the PBS and discharge the solution.
  b. Detach cells by adding 5 mL Trypsin-EDTA (0.25%; Ethylene diamine tetra acetic acid) to the flask and return the flaks to 37°C for 5 min. Deactivate trypsin by adding 5 mL DMEM #1 medium. Aspirate the cell suspension and transfer to a 15 mL falcon tube. Add 5 mL DMEM #1 to the cells.
  c. Centrifuge the cell suspension at 500 × *g*, at 18°C–22°C, and for 5 min.
  d. Resuspend the cells in 10 mL DMEM #1 medium and split the cells to the required number of new cell culture flasks at a 1:10 ratio in DMEM #1 medium (e.g., 1 mL cell suspension and 9 mL medium).
  e. Excess cells may be frozen down for later use.
    i. Briefly, count the number of viable cells using the trypan blue staining method and microscopy, spin the cells as described above, discharge the supernatant, and resuspend the cells in pre-cooled (4°C) freezing medium to yield ca. 5×10^6^ cells/mL.
    ii. Add 1 mL of the cell suspension to cryo tubes, and place the tubes in a pre-cooled (4°C) freezing container. Place the container in a freezer (−80°C) and leave for 24 h. For long term storage, transfer tubes to a liquid nitrogen tank.

### Preparation of reagents

**Timing: 15 min**

The following procedures including the open handling of chemicals is performed in a chemical safety hood with ventilation and by wearing personnel protection equipment such as lab goggles, lab gloves, and lab coat. Make sure to have spill kits available in case of spillage. Of note, crystal blue makes very stable stains if spilled on surfaces and clothing. Clean immediately by first using dry tissues to extract most of the solution. Then use wet tissues with ethanol to clean the rest.

5. Add 13.51 mL formaldehyde solution (37%) into a sterile glass flask and dilute with 36.49 mL ddH_2_O to yield a 10% solution. Label the flask with “Date of preparation”, store at 18°C–22°C, and use within one month.
6. Mix 25 mL Gram-Hucker’s crystal violet oxalate solution with an equal volume of ddH_2_O using a sterile glass flask. Label the flask with “Date of preparation”, store at 18°C–22°C and protected from light, and use within six months.

## Key resources table

**Table:** 

REAGENT or RESOURCE	SOURCE	IDENTIFIER
**Bacterial and virus strains**		

SARS-CoV-2	Volker Thiel, University of Bern	www.nature.com/articles/s41586-020-2294-9

**Biological samples**		

Patient-derived serum	University Hospital Zurich	n/a

**Chemicals, peptides, and recombinant proteins**		

DMEM (Dulbecco`s Modified Eagle`s Medium; high glucose)	Gibco	11995065
MEM NEAA (non-essential amino acids)	Gibco	11140050
HEPES (1M; N-2-hydroxyethylpiperazine-N-2-ethane sulfonic acid)	Gibco	15630–080
Normocin™ (antimicrobial reagent)	InvivoGen	ant-nr-2
Trypsin-EDTA (0.25%; ethylenediaminetetraacetic acid)	Gibco	25200–056
FBS (Fetal Bovine Serum "GOLD" Heat Inactivated)	PAA	A15-152
Formaldehyde solution (37%)	Sigma	F8775-500
Gram-Hucker's crystal violet oxalate solution	PanReac AppliChem	252532.1211
PBS (phosphate-buffered saline; pH 7.4)	Gibco	10010–015
Gigasept® AF for decontamination	Schülke	180832
Trypan blue stain (0.4%)	Gibco	15250–061
DMSO (dimethyl sulfoxide)	Sigma	276855

**Experimental models: cell lines**		

African green monkey: Vero	ATCC	CCL-81

**Software and algorithms**		

GraphPad Prism 8.0 or higher	GraphPad Software Inc.	www.graphpad.com

**Other**		

Pipetboy acu 2	Integra Biosciences	612–0926
Micropipette (2–20 μL)	Eppendorf	E-1863
Multichannel pipette; Fin Tip®, 12-channel (30–300 μL)	Thermo Fisher	11847421
Pipette tips 20 μL; TipOne®	STARLAB	S1123-1810
Pipette tips 300 μL; Finntip Flex 300	Thermo Fisher	11852703
Serological pipettes 10 mL	Greiner	612–3325
Serological pipettes 25 mL	Greiner	612–1017
Falcon™ 15 mL tubes	Falcon	10263041
Falcon™ 50 mL tubes	Falcon	10788561
Corning™ Costar™ Reagent reservoir	Thermo Fisher	10261391
Corning™ Falcon™ cell culture flasks, T-150	Thermo Fisher	734–0050
CELLSTAR® 96-well cell culture plates, flat bottom	Greiner	391–3325
CELLSTAR® 96-well plate culture plates, round bottom	Greiner	392–0019
Nalgene® Cryo Tubes	Thermo Fisher	V5007-500EA
Corning® CoolCell LX Cell Freezing Container	Thermo Fisher	CLS432002
500 mL Vacuum filtration “rapid”-filtermax units (0.2 μm)	TPP	99500
Thermoblock	Eppendorf	Thermomixer C
CO_2_ incubator	Thermo Scientific	BBD 6220
Microcentrifuge	Eppendorf	5418 R
Benchtop centrifuge	Eppendorf	5810 R
Neubauer cell chamber (haematocytometer)	VWR	631–1112
Light microscope	Zeiss	Axiovert 25
Digital camera Lumix	Panasonic	DC-TZ202
Light board	Kaiser	slimlite plano

## Materials and equipment

**Table:** DMEM #1 cell culture medium

Reagent	Final concentration	Amount
DMEM (high glucose)	n/a	464 mL
FBS (heat-inactivated)	5%	25 mL
MEM non-essential amino acids	1%	5 mL
HEPES (1M)	1%	5 mL
Normocin™	0.2%	1 mL
**Total**	**n/a**	**500 mL**

Store at 2°C–8°C and for maximum of one month.

**Table:** DMEM #2 medium for serum dilution

Reagent	Final concentration	Amount
DMEM (high glucose)	n/a	496 mL
FBS (heat-inactivated)	1%	5 mL
Normocin™	0.2%	1 mL
**Total**	**n/a**	**500 mL**

Store at 2°C–8°C and for maximum of one month.

**Table:** DMEM #3 medium for use in culture with virus

Reagent	Final concentration	Amount
DMEM (high glucose)	n/a	474 mL
FBS (heat-inactivated)	1%	10 mL
MEM non-essential amino acids	2%	10 mL
HEPES (1M)	2%	5 mL
Normocin™	0.2%	1 mL
**Total**	**n/a**	**500 mL**

Store at 2°C–8°C and for maximum of one month.

**Table:** Freezing medium for cells

Reagent	Final concentration	Amount
FBS (heat-inactivated)	n/a	45 mL
DMSO	10%	5 mL
**Total**	**n/a**	**50 mL**

Store at -20°C. After thawing, store at 2°C–8°C and use within five days.

***Note:*** Process the medium immediately into sterile containers by membrane filtration with a 0.2 μm sterile filters using a positive pressure system. Store all media at 4°C.

## Step-by-step method details

### Seeding of Vero cells

**Timing: 30 min work, 24 h incubation**

Vero cells express the ACE2 receptor, which is targeted by SARS-CoV-2 via the virus’s spike proteins to infect cells (Fard et al., 2021). Here, we describe the infection of Vero cells with SARS-CoV-2.

1. Use Vero cells that are 80%–100% confluent, i.e., adherent cells are taking up 80%–100% of the surface of the flask. Aspirate and discard culture medium and wash the cells gently with PBS as described in step 4 above.
2. Detach the cells from the flask.
  a. Add 5 mL Trypsin-EDTA (0.25%) to the flask and return the flask to 37°C for 5 min incubation.
  b. Deactivate Trypsin-EDTA by adding 5 mL DMEM #1 medium. Aspirate the cell suspension and transfer to a 15 mL falcon tube.
  c. Add 5 mL DMEM #1 to the cells. Centrifuge cell suspension at 500 × *g*, at 18°C–22°C, and for 5 min.
3. Resuspend the cells in 5 mL DMEM #1 medium, count the cells using the trypan blue staining method and microscopy, and set cell concentration to 1×10^5^ cells/mL.
4. Seed 1×10^4^ cells (100 μL) per well in flat-bottom 96-well cell culture plates and incubate the plates at 37°C and 5% CO_2_ for 24 h.

**CRITICAL:** The number of seeded Vero cells is critical and can be used as a modulator for the experiment (cf. troubleshooting, problem 1 below). The cells should not be more than 80% confluent after 24 h incubation in the 96-well plate, so that control wells do not overgrowth during the later incubation step (3 days). Otherwise, overgrown cells start to detach and the distinction between cytopathic effect and cell detachment due to overgrowth becomes difficult or impossible.

### Preparation of serum samples and infection

**Timing: 3 h work, 3 days incubation**

The assay allows the detection of neutralizing antibodies in serum of convalescent or vaccinated persons. The sera are mixed with live virus, and the presence of neutralization antibodies will prevent viral infection of Vero cells. The virus neutralization can be measured by means of reduced visual cytopathic effect of virus on Vero cells. As non-qualified human sera may potentially contain human pathogens, their open handling of should take place in a biosafety hood with laminar air flow. The serum used in the following study was from the serum collection of the University Hospital Zurich (USZ) Dermatology Biobank, were collected under the General Ethical Consent of USZ, and they were stored at −20°C.

5. Pre-treat the serum samples at 56°C and for 30 min in Eppendorf tubes in a Thermoblock.

***Note:*** The heat treatment destroys complement proteins and has been recommended for virus neutralization assays. However, and as the result will demonstrate, the heat-treatment of sera is not mandatory. The current assay works with either heat-treated or non-treated sera (cf. Figure 6 below).

**Figure 1:**
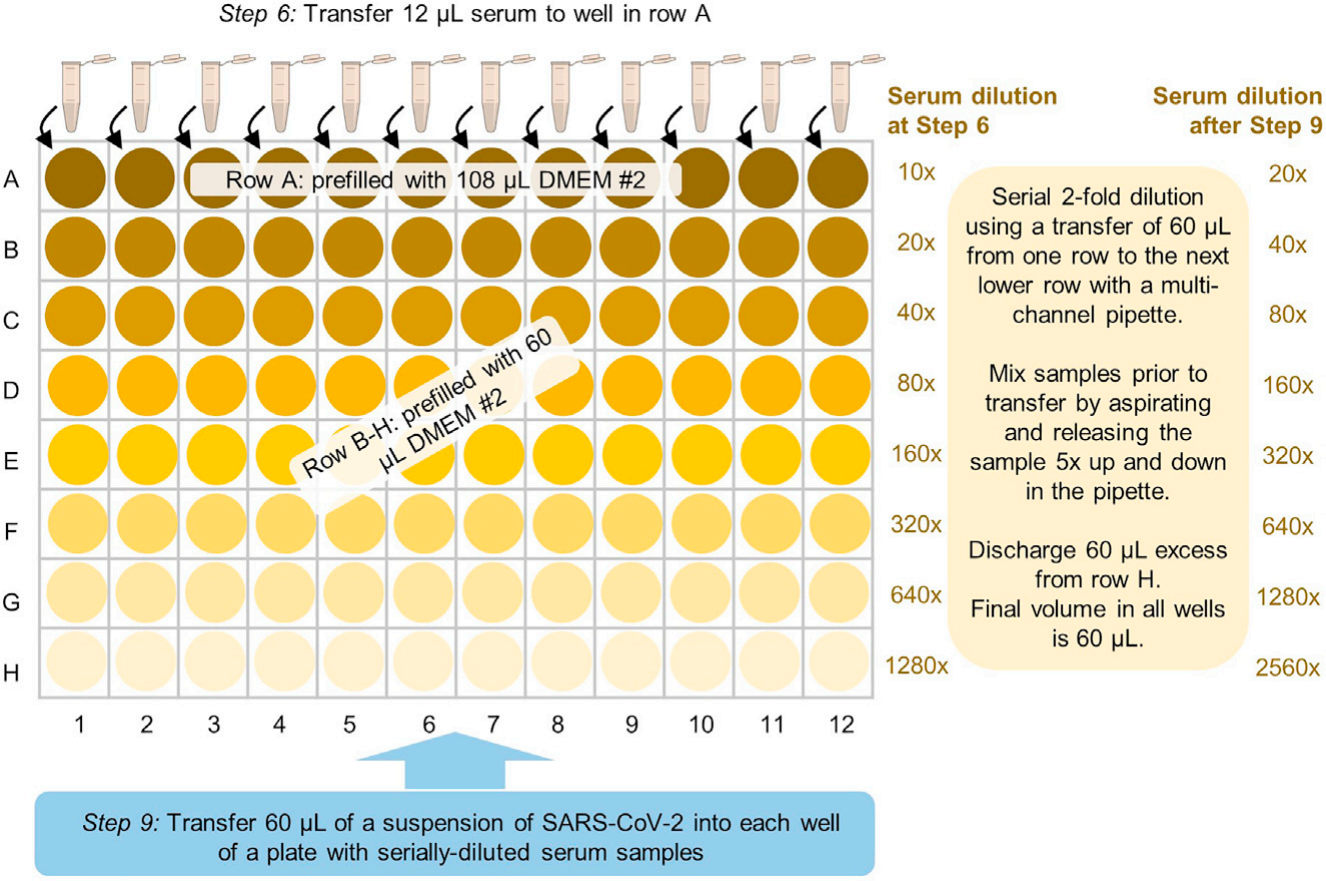
Preparation of 96-well plate for serum dilution and virus neutralization Serum samples in tubes are added to 96-well plates with culture medium (DMEM #2). Two-fold serial dilutions thereof are made using a multichannel pipette, so that samples ranges from a 10-fold to a 1280-fold dilution prior to addition of virus. As the volumes of serum solution and virus solution are the same, there is an additional two-fold dilution of the serum samples upon adding virus (new dilution: 20-fold–2560-fold).

***Note:*** Different starting dilution may be chosen, e.g., D5 or D100, and different serial dilutions variants may be used, e.g., 3-, 5- or 10-fold dilutions, depending on expected strength of the serum neutralization, and the expected range of neutralization titers between serum samples.

6. Prepare dilutions of serum samples.
  a. Use a multichannel pipette to prefill 96-well round-bottom plates with 108 μL DMEM #2 medium in row A and 60 μL DMEM #2 medium in rows B-H.
  b. Make a primary 10-fold dilution of serum samples by transferring 12 μL serum to each well of row A. Mix the samples in row A by aspirating and releasing 60 μL of the samples five times using the multichannel pipette.
  c. Make 2-fold dilutions of the serum samples by transferring 60 μL of the serum solution in row A to the medium-containing row B. Replace the pipette tips and mix the samples in row B by aspirating and releasing 60 μL as above.
  d. Proceed the serial 2-fold dilution down the plate for a total of eight dilutions (D10, D20, D40, D80, D160, D320, D640, and D1280), and replace the tips between each row.
  e. Discard 60 μL from the last row so that the final volume of each well is 60 μL. Figure 1 illustrates the plate preparation procedure.

7. Seal the 96-well plates with the diluted serum samples with a lid and a plastic foil, and store the plates at 4°C until the next steps.

**CRITICAL:** Each step from now on must be performed in a BSL3 laboratory according to internal guidelines. Only trained and experienced personnel should handle life virus to avoid non-intended contamination and infections with SARS-CoV-2, which is stored at −20°C within the BSL3 lab. In the BSL3 lab, it is mandatory to wear personal protective equipment, and effort should be put into organizing the work within the lab and within the biosafety hood. In the described protocol, two persons were always present in the BSL3 lab on days 1 and 5. This enabled one person to work efficiently in the biosafety hood while the second person provided materials. Moreover, it is often a safety guideline to always have at least two persons in a BSL3 lab.

8. Thaw a frozen aliquot of the virus suspension by leaving it in the biosafety hood for approximately 15 min. Prepare the targeted concentration (typically in plaque-forming units per mL; in the example below, with 100 PFU infection, a concentration of 1666.7 PFU/mL is required) of the virus by pipetting an accurate aliquot thereof into a 50 mL falcon tube prefilled with DMEM #3. Transfer the whole virus suspension into a reagent reservoir.
9. Aspirate 60 μL of the virus suspension using a multichannel pipette and transfer the suspension to the row A on the plates with the diluted serum samples (from step 7). Repeat the procedure for the rows B-H, and replace the pipette tips between each row. Figure 1 illustrates the plate preparation procedure.
10. Incubate the plates at 37°C and 5% CO_2_ for 1 h.
11. Infect cells by transferring 100 μL of the serum-virus solution to each well of the plates with the adherent Vero cells (from step 4); make sure to re-suspend the serum-virus samples by pipetting up and down twice before the transfer. Label the plates and lids for recognition.
12. Incubate the plates at 37°C in a humidified and 5% CO_2_ atmosphere for 3 days.

**CRITICAL:** Include non-infected controls and controls with naïve non-neutralizing serum. This is important as to be able to distinguish between true cytopathic effect and other unforeseen cell-culture effects such as mechanical disruption of the cell layer that may influence the assessment of neutralization.

***Note:*** The exact incubation time depends on the starting number of Vero cells, viral titers, and the strength of neutralization. It is recommended to try different incubation times (typically 2–4 days). Moreover, the optimal number of viral particles used may depend on the particular virus isolation serotype and the genetic variant of the viral strains. As a rule, the cytopathic effect increases with time and initial viral dose. The current protocol with the given SARS-CoV-2 strain was optimized for infections with 100 PFU per well, hence, a virus suspension containing 1666.7 PFU/ml was prepared. The concentration of cytopathic virus is by standard defined by their property to create plaques (holes) in a confluent cell layer of cells when these are overlaid with a gel (Baer and Kell-Hall, 2014). The virus load should be optimized in each lab and for each viral strain.

### Staining of infected cells

**Timing: 2 h**

**Figure 2:**
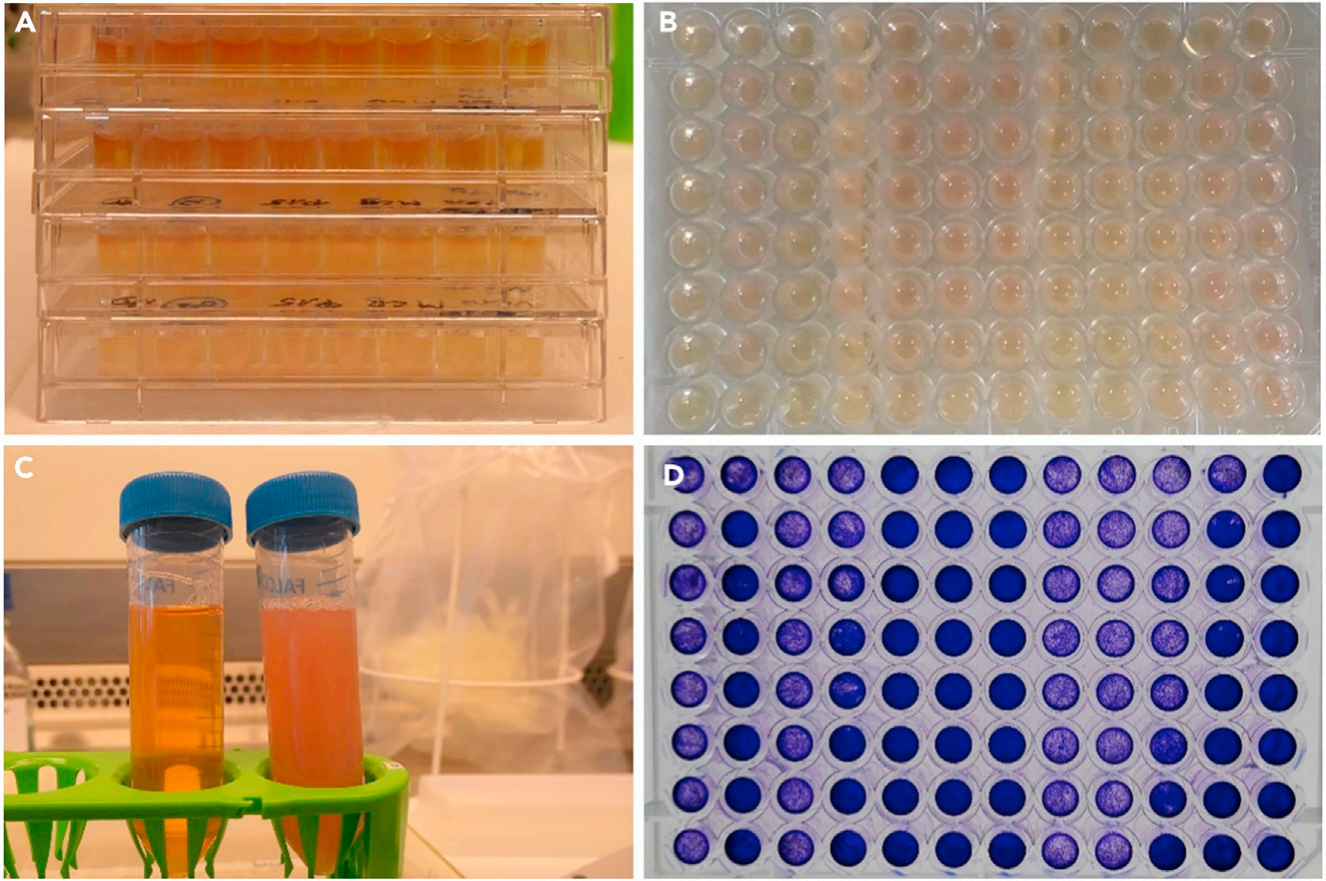
In-process visual control of infections and neutralization After 3 days of incubation of Vero cells with SASR-COV-2 virus, visual control of cultures will enable to distinguish infection from non-infections (or successful neutralization). (A) Side view of four plates prior to inactivation of virus with formaldehyde. The upper two plates show wells with healthy cells (no infection) as indicated by the red color of the culture, while the lower two plates show infected cells as indicated by the yellow color of the cultures. (B) Culture after inactivation with formaldehyde (step 10). Wells with infected cells are more yellow. Wells with non-infected cells are redder. (C) The discharged culture wash (step 11) of plates with healthy cells is clear (left tube), as cells maintain adhered on the plate. The discharged culture wash of infected cells is more turbid (right tube) due to detachment of cell from plate. (D) The same plate as in B after staining (steps 12–15). Note that yellowish wells in B show non-confluence cell layers (cytotoxicity) in D, while the reddish well is B show confluent cell layers in D.

After three days of incubation, several rounds of infection should have produced visible cytopathic effects (Figure 2A). Remaining live virus is neutralized by formaldehyde. Vero cell detachment can be observed directly on plate (Figure 2B) or under microscope, but to increase contrast, the cells are stained with crystal violet.

13. Add 10% formaldehyde solution to a reagent reservoir (10 mL per plate is required) using a serological pipette. By using a multichannel pipette, transfer 100 μL/well to the infected plates in order to fixate cells and to inactivate virus.
14. Incubate the plates at 18°C–22°C for 15 min.
15. Discard supernatant into a reagent reservoir by using a multichannel pipette. Wash the plates with 100 μL/well PBS and discard wash solution, again using the multichannel pipette. Of note, cytotoxicity can be observed by having more cells in the wash solution (Figure 2C). These are cells that were detached from the plate due to infection.
16. To stain cells, add 50 μL/well of 1% Gram Hucker’s crystal violet solution by using a multichannel pipette.
17. Incubate the plates at 18°C–22°C for 15 min.
18. Remove staining solution using a multichannel pipette and discharge waste into a reagent reservoir. Wash the plates with 100 μL/well PBS, and discard wash solution into the reagent reservoir.
19. Remove residual washing solution by tapping the plates top-down and gently on a tissue paper.
20. Disinfect the plates with lids using 70% ethanol in water. Only now, the plates can be taken out of the biosafety hood.
21. Collect all the liquid wastes into 50 mL falcons and close with lids.
22. Place the stained plates upside down on a luminous board, a light table, or similar. Make photographs of the plates from underneath (Figure 2D) as well as one matched photograph of the labeled lid for identification of the plate organization.
23. Analyze cytopathic effect and note the serum dilutions where 50% or more of the cell layer is harmed or detached.
24. Dry plates top-down at 18°C–22°C overnight or longer.

***Note:*** All liquid waste from step 8–15 is collected in Gigasept® for decontamination and then discharged in bags together with the solid waste. Finally, all waste is autoclaved. The waste management in different BSL3 labs may vary, and typically defined by the pathogen (here, SARS-CoV-2), and not by the chemicals. The BSL3 waste management is always strongly regulated by law and typically ISO certified.

## Expected outcomes

This protocol describes the determination of SARS-CoV-2 neutralization titers of serum from convalescent patients or vaccinated individuals. The neutralization effect can be estimated by the level of cytopathic effect in SARS-CoV-2-infected Vero cells for a defined dilution. Compared to the often-used plaque-forming unit (PFU) protocols with 6- or 12-well plates, the current assay in 96-well format allows simultaneous screening of dozens of serum samples in parallel. Compared to the TCID_50_ assay, all tests can be done with the same viral load, while TCID_50_ requires virus dose optimization for each sample.

## Quantification and statistical analysis

**Figure 3:**
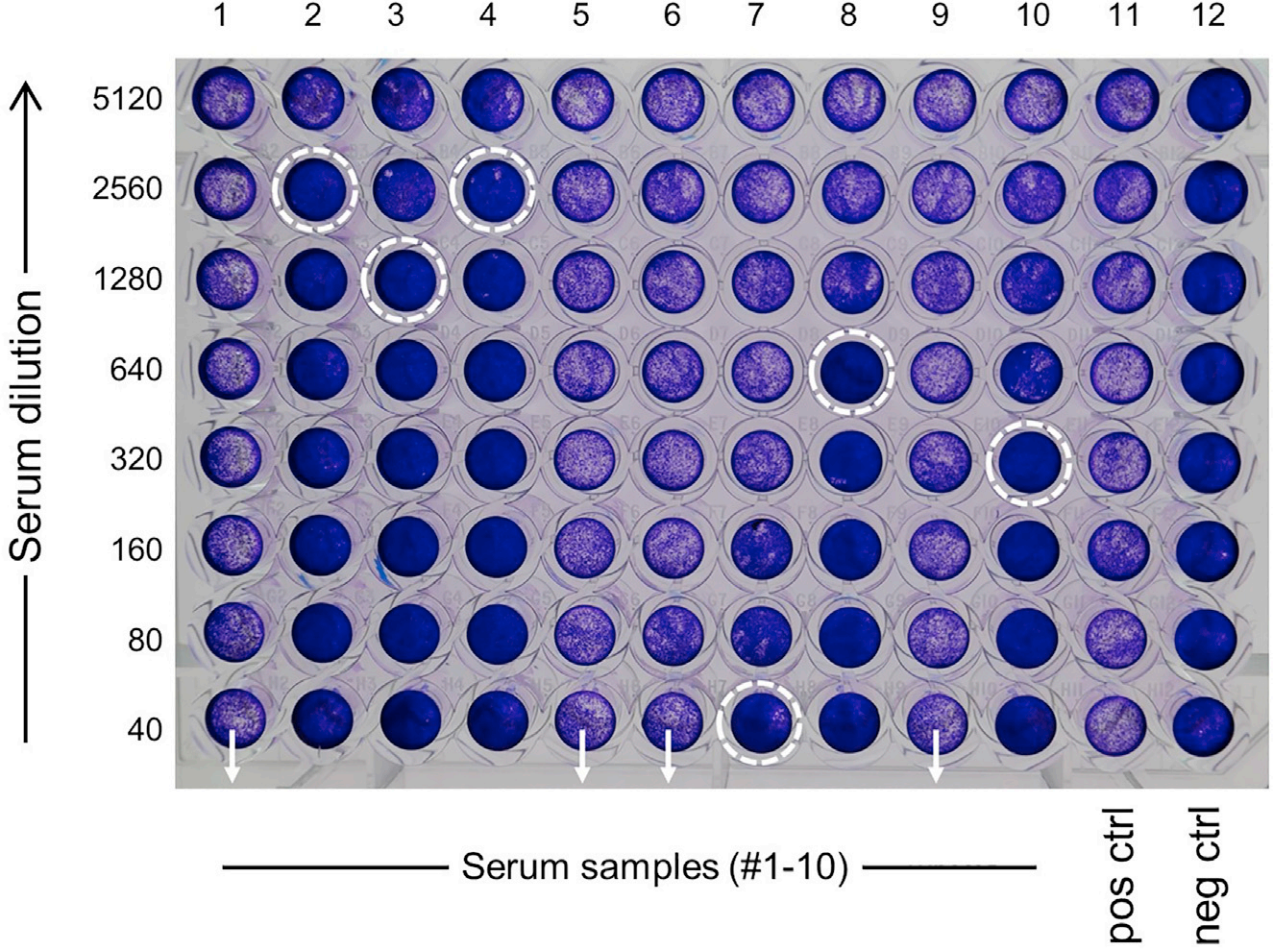
Example of stained plate with visible cytopathic effect Columns 1–10 depict samples from vaccinated persons, column 11 the positive control (cells, virus, no serum), and column 12 the negative control (cells, no virus, no serum). The serum samples are two-fold diluted, with the highest serum concentration in the bottom rows. The arrows for samples # 1, 5, 6, and 9 indicate that the neutralization titers were below detection limit (lowest serum dilution). The white hatched circles indicate the neutralization titer of the different sample.

**Figure 4:**
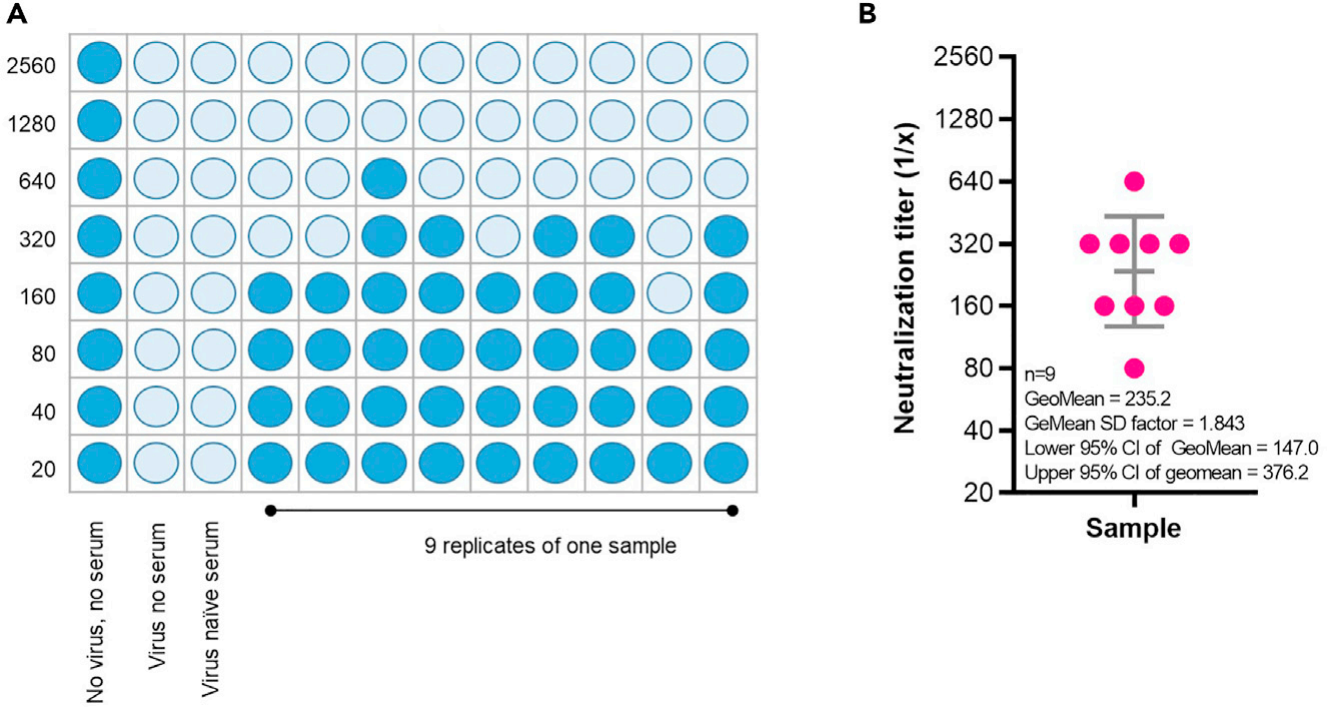
Illustrated analysis of virus neutralization titer (A) The plate shows a thought neutralization assay with serum samples 2-fold diluted from 1/20 (bottom row) to 1/2560 (upper row). The first three columns from left are controls, and the last 9 columns are replicates of a samples (or different samples). The dark blue wells of the plate show intact cell cultures (confluent), while the light blue wells illustrate detached cultures, hence, infected cells. The last dark blue well before a light blue well appears, is defined as the neutralization titer. (B) Graph shows the scatter plot of the nice serum samples in A and the geometric mean and 95% confidential intervals of the samples as calculated in Graph Pad Prism version 8.0.

**Figure 5:**
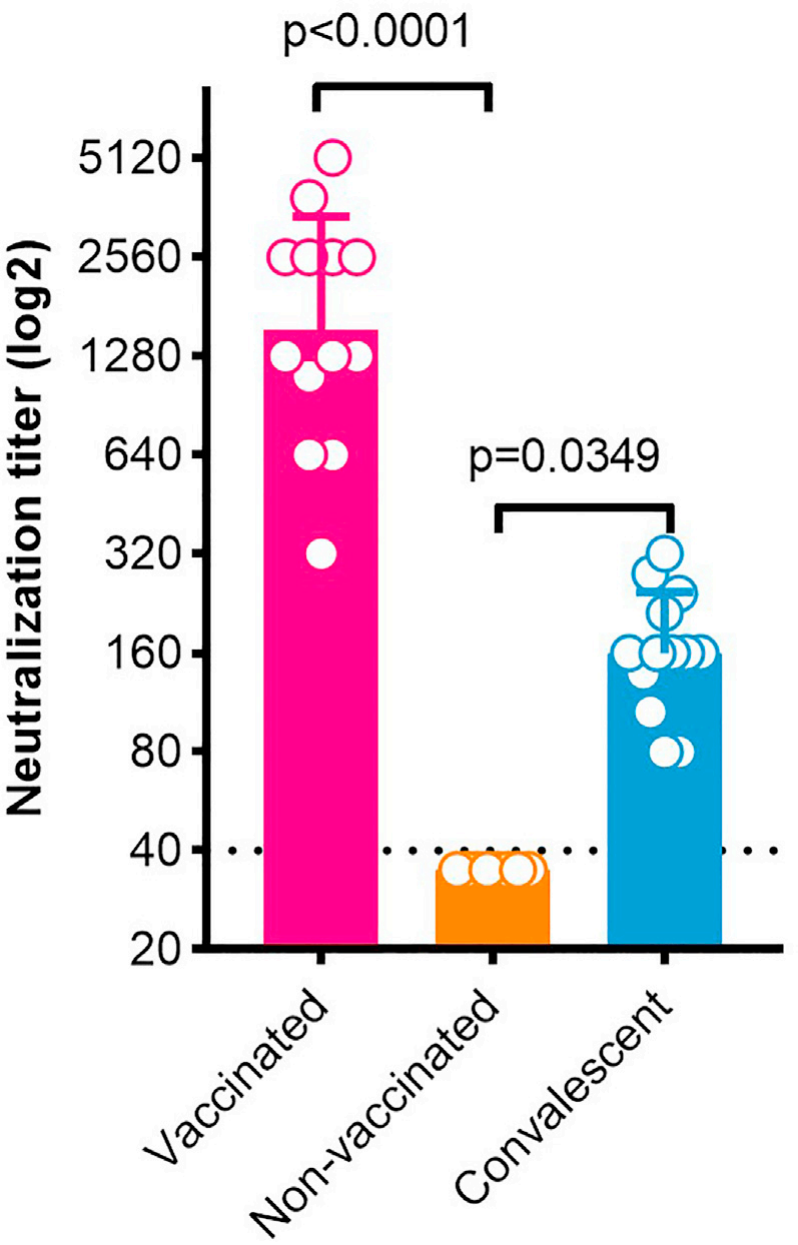
Example of quantification of virus neutralization in serum samples from vaccinated, non-vaccinated (naïve) and from convalescent individuals Thirty four serum samples from persons that had been vaccinated against Covid-19, recovered from a confirmed Covid-19 disease, or from healthy and non-vaccinated individuals were tested for neutralization against SARS-CoV-2. The data are analyzed by Kruskal-Wallis test for non-parametric data and multiple comparisons were corrected using the Dunn’s test (from Graph Pad Prism 8.0). Geometric means with geometric standard deviations are shown.

Based on the stained plates, a neutralization titer for each sample is determined. Figure 3 shows a plate with 10 serum samples as well as positive and negative controls. When testing replicates of the same samples, the geometric mean of the replicates should be calculated, since the data are not following a normal but a log distribution. Figure 4 illustrates a plate scheme (Figure 4A) and the analysis of the plate (Figure 4B). Figure 5 shows the summarized analysis of virus neutralization in serum samples from vaccinated and non-vaccinated individuals, and in sera from SARS-CoV-2-infected, but recovered individuals.

## Limitations

The virus used in the current assay is a synthetically reconstructed variant of SARS-CoV-2 (Thao et al., 2020), which again is based on an early isolated SARS-CoV-2 strain from Wuhan, China (Zhu et al., 2020). When using other SARS-CoV-2 variants, different isolates or mutated strains, which may differ in virulence on Vero cells or other cell lines applied, the conditions for culturing the cells may need to be adjusted. Furthermore, human cell lines may be more suitable for a more accurate depiction of SARS-CoV-2 infection. Nevertheless, it is essential that the chosen cell type is adherent on culture plates during the whole experiment. The protocol works best with fresh Vero cells from low passage numbers. Late passages (>30) may detach from culture plates even in absence of live virus, especially when using long incubation time (3 days or more). Such an effect will make the detection of cytopathic effects non-distinguishable from cell detachment not associated with an infection.

**Figure 6:**
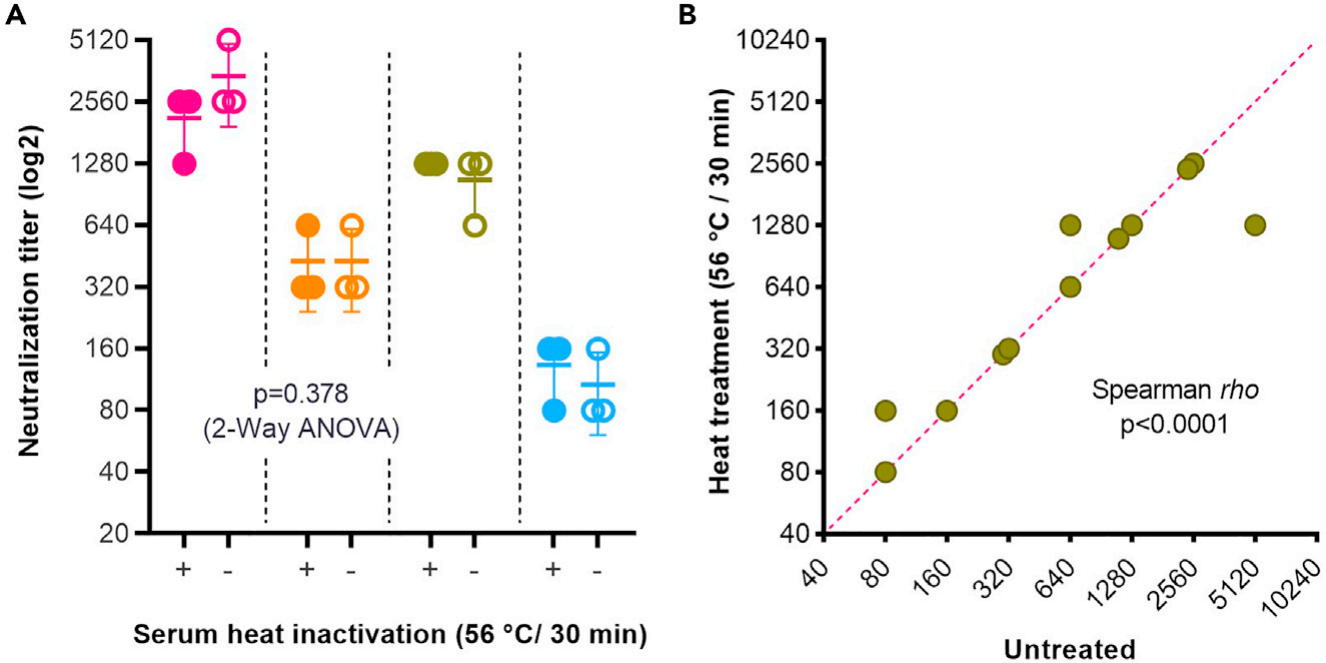
The quality of virus neutralization does not depend upon heat-inactivation of serum Serum samples from four convalescent persons split in two aliquots each. One aliquot was heat treated at 56°C for 30 min to inactivate complement, and the other aliquot was left untreated. (A) The samples were then tested for SARS-CoV-2 neutralization in triplicates. The data were statistically analyzed by 2-way ANOVA using heat treatment (yes or no) and serum sample source (person) as factors. The p-value refers to the effect of the heat treatment. Geometric means with geometric standard deviations are shown. (B) Spearmen’s correlation plot and statistical analysis (two tailed) of heat treated versus non-treated serum samples.

As mentioned above, heat treatment of sera has been recommended for virus neutralization assays. In the current assay, the heat-treatment of sera was not mandatory (Figure 6). However, this may be limited to Vero cells, for which reason the effect of heat-inactivation of complement in serum should be tested when changing to another cell type.

## Troubleshooting

### Problem 1

Cells do not grow enough or overgrow on plate for infection.

### Potential solution

Try different cell numbers for the 24 h incubation period before addition of virus. Approximately 10,000 Vero cells per well in a 96-well plate are sufficient for a confluent monolayer after 4 days of incubation. Vero cells tend to lose adherence capacity with increasing passage number. In this case, try using new cells from a lower passage. To find the optimal cell number on the 96-well plate, seed different cell numbers (e.g., 5,000, 10,000, 20,000, or 40,000 cells) for different time periods (24, 48, 72, or 96 h) with and without viral infection, and stain as described in the protocol. Use the combination of cell number and incubation period that produces a smooth and regularly stained layer of cells for non-infected cultures and a clearly visible cytopathic effect upon infection.

### Problem 2

The Vero cells are growing slowly.

### Potential solution

Vero cells recover slowly after freezing; and it may take a week or more before the cells are ready to be passaged. After 2–3 passages, the Vero cells reach their normal growth rate. When you plan an experiment, the slower growth of freshly thawed cells should be taken into consideration.

### Problem 3

Cytopathic effect on the infected plate is too weak or to strong.

### Potential solution

If the cytopathic effect is too weak, try to increase the number of viral particles or the incubation time. If the cytopathic effect is too strong, reduce the number of viral particles or the infection time. Using the protocol described here, the cytopathic effect starts with singles plaques after approximately one day of incubation. Full cell detachment is visible after two days.

### Problem 4

The cytopathic property of the virus is decreasing from one experiment to another.

### Potential solution

The viability of SARS-CoV-2 is reduced when being left too long in solution without cells or upon cycles of freezing and thawing. Hence, do not re-freeze aliquots of virus for use in a later experiment. Upon arrival of virus, make aliquots that are appropriate for the size of the experiments performed, and re-establish infecting dose for each new batch of virus.

### Problem 5

Difficulties in identifying and analyzing cytopathic effects.

### Potential solution

Counting by eye may be less error-prone than analyzing an image of a plate. Try analyzing cytopathic effect by directly on the stained plate. If contrast is weak, one may use a light board with a brighter or stronger light. Alternatively, if analyzing images of plates with image software, one can adjust brightness, color and contrast to improve analysis.

## Resource availability

### Lead contact

Further information and requests for resources and reagents should be directed to and will be fulfilled by the lead contact, Pål Johansen (pal.johansen@usz.ch).

### Materials availability

This study did not generate unique reagents.

## Data Availability

This study did not generate unique datasets or code.
